# Supplementary material for: Comparative Genomics Revealed a Potential Threat of Aeromonas rivipollensis G87 Strain and Its Antibiotic Resistance
Source: Antibiotics (Basel). 2023 Jan 9;12(1):131. doi: 10.3390/antibiotics12010131 (PMC9855013; doi:10.3390/antibiotics12010131)
Supplement: Supplementary file 1 [file antibiotics-12-00131-s001.zip › antibiotics-2091250-supplementary.pdf]

Supplementary Materials:

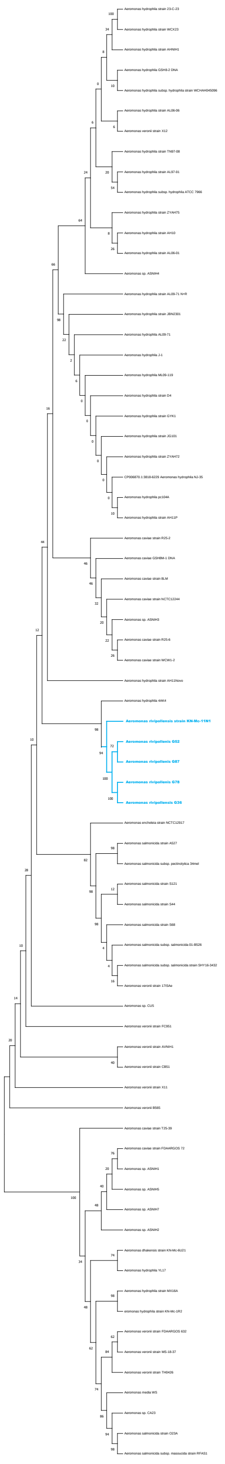

Figure S1. Phylogenetic analysis of the *Aeromonas* species isolates using the *gyrB*. The 4 sequenced *A. rivipollensis* are highlighted in blue as well as the reference strain KN-Mc-11-N1. Bootstrap value of 1000 was used to construct the tree.

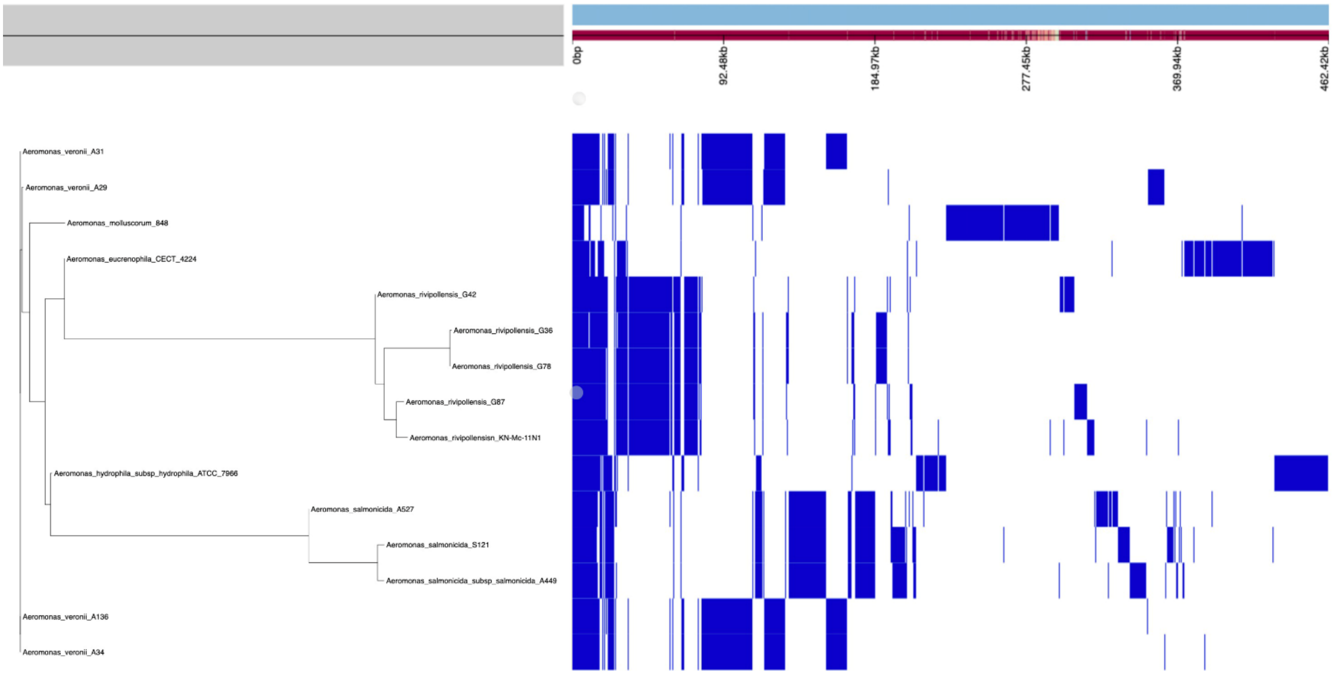

Figure S2. Pangenome genome of the 15 *Aeromonas* spp. isolates showing the core, shell, and cloud genes. A total of 23119 CDS genes was used to construct the pangenome phylogenetic tree generated using Phandango. The colour coded blue represents compared coding genes amongst the genomes.

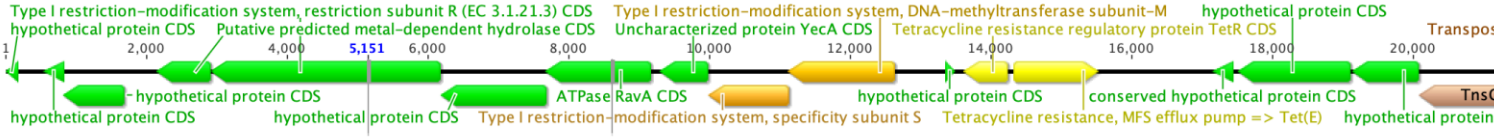

Figure S3. Gene organization of tetracycline resistance genes (yellow) with transposase *ISAs31* and transposition protein *TnsC* (brown) as well as Type 1 restriction recognition sites subunits (orange) that drives horizontal gene transfer determined in *A. rivipollensis* G87 genome. The CDS were annotated using prokka and visualized on Geneious version 9.0.5.
